# Systemic, Ocular and Genetic Risk Factors for Age-related Macular Degeneration and Polypoidal Choroidal Vasculopathy in Singaporeans

**DOI:** 10.1038/srep41386

**Published:** 2017-01-25

**Authors:** Chui Ming Gemmy Cheung, Augustinus Laude, Ian Yeo, Shu-Pei Tan, Qiao Fan, Ranjana Mathur, Shu Yen Lee, Choi Mun Chan, Gavin Tan, Tock Han Lim, Ching-Yu Cheng, Tien Yin Wong

**Affiliations:** 1 Singapore National Eye Centre, Singapore; 2 Singapore Eye Research Institute, Singapore; 3 Department of Ophthalmology, Yong Loo Lin School of Medicine, National University of Singapore, Singapore; 4 Department of Ophthalmology, National Healthcare Group Eye Institute, Tan Tock Seng Hospital, Singapore

## Abstract

To examine the association of systemic, ocular and genetic risk factors in neovascular age-related macular degeneration (nAMD) in a large cohort of Asian patients, and to further compare risk factors between those with typical AMD and polypoidal choroidal vasculoapthy (PCV) subtypes. We recruited 456 cases and 1,824 controls matched for age, gender and ethnicity. Data on systemic and ocular risk factors were collected on questionnaires. In a subgroup of subjects, we included genetic data on four AMD-associated single nucleotide polymorphisms (SNPs). Risk factors for nAMD and subtypes were analyzed. Systemic risk factors for nAMD included older age, male gender, higher BMI and higher HDL-cholesterol. Ocular risk factors included pseudophakic and shorter axial length. Risk factors common to both typical AMD and PCV subtypes included age, BMI and HDL-cholesterol. Shorter axial length was only associated with PCV, while male gender and pseudophakia were only associated with typical AMD. In the subgroup with genotype data, *ARMS2* rs10490924 and CFH rs800292 were associated with nAMD. None of the risk factors were significantly different between PCV and typical AMD. Systemic, ocular and genetic risk factors were largely similar for typical AMD and PCV subtypes in this Asian population based in Singapore.

Age-related macular degeneration (AMD), particularly neovascular AMD (nAMD), is one of the most common causes of blindness worldwide in individuals older than 60 years^1^,^2^. The prevalence rate of AMD in populations with European ancestry has been reported in many previous studies^3^,^4^,^5^. Racial and ethnic differences in prevalence have been suggested in some early studies^3^,^6^. More recently, however, several population studies in Asians have added to our understanding of the prevalence of AMD in Asian populations, and suggest that prevalence of late AMD^7^,^8^,^9^,^10^,^11^,^12^, in particular, is largely similar between Asian and European populations^13^. We have reported similar prevalence of AMD between Chinese, Malays and Indians residing in Singapore, and also between Indians residing in Singapore^14^,^15^ and those residing in Central India^16^.

A wide range of risk factors for AMD have been reported in many studies in patients with European ancestry^17^,^18^,^19^,^20^,^21^,^22^,^23^. Older age and smoking have been consistently described as risk factors for AMD. A meta-analysis of 73 risk factors previously reported mostly in Europeans showed strong and consistent associations with increasing age, current cigarette smoking, previous cataract surgery and a family history of AMD^24^. In addition, higher body mass index, a history of cardiovascular disease, hypertension and raised fibrinogen levels were found to have moderate and consistent associations with AMD. Associations between changes in cholesterol levels and AMD have been less consistent. Pooled data from three population studies in patients with European ancestry showed that total serum cholesterol was inversely associated with incident nAMD^25^.

The risk factors for AMD in Asians have been less well studied, with three major limitations. First, most population-based studies of AMD have focused on early AMD (e.g., drusen, retinal pigment epithelial changes). With respect to late AMD, we previously reported that late AMD (nAMD and geographic atrophy) was associated with age, smoking >5 packs per week and chronic kidney disease in the population-based Singapore Epidemiology of Eye Disease study^14^. Smoking were also reported as a significant risk factor for the development of late AMD in two population-based studies in Hisayama^10^ and Funagata^9^ in Japan. Other risk factors that have been reported inconsistently for late AMD include hypertension, lipid disturbance and hyperopia^8^,^26^,^27^. However, systemic parameters of gender, body mass index, diabetes mellitus, or hypertension were not associated with late AMD in the Beijing Eye study or the Shihpai Eye study, both in subjects with Chinese ethnicity^7^,^8^,^11^,^12^. However, the small number of subjects with late AMD in population studies is a major limitation in the identification of risk factors.

Second, nAMD in Asians is also often divided into typical AMD with clinical features similar to those seen in Western populations, and polypoidal choroidal vasculopathy (PCV)^1^,^28^,^29^. Whether these two subtypes are associated with different risk factors remains unclear. Due to the need for detailed angiographic differentiation between these two subtypes, there has been little data from population studies in this area. Limited comparative studies using clinical cohorts have reported that background factors were similar, although diabetes mellitus and end-stage renal disease appear to be more prevalent in patients with typical AMD than in those with PCV in some studies^30^,^31^,^32^.

Finally, genetic factors, most strongly attributed to single nucleotide polymorphisms (SNPs) in the complement factor H (*CFH*) gene and the *ARMS2/HTRA1* loci, have been estimated to account for approximately half of the heritability of AMD in Western cohorts^33^,^34^,^35^. In Asians, the Genetics in AMD in Asians (GAMA) consortium reported associations of *CFH* and *ARMS2/HTRA1* with Asian AMD in genome-wide association study. In addition, a novel missense mutation in the cholesteryl ester transfer protein (*CETP*) was reported^36^. Whether these genetic variants confer similar risk for typical AMD and PCV subtypes remains controversial, but there has been increasing interest in a possible association between genes in the HDL metabolic pathway and PCV^37^,^38^.

The prevalence of AMD in Asia is expected to increase significantly due to a combination of population growth, aging population and changes in lifestyle factors. Asia accounts for over 60% of the world population and is expected to see the largest projected number of cases of AMD^1^,^14^. Therefore there is a pressing need to better understand the systemic, genetic and ocular risk factors for AMD in Asians. To this end, we designed the Asian AMD Phenotyping Study in 2010 with the aim to study the clinical features and risk factors of Asian nAMD, including further subtyping into typical AMD and PCV according to angiographic features in every case. To facilitate comparison of potential risk factors, the Asian AMD Phenotyping study adopted methods modelled after the Singapore Epidemiology of Eye Disease program which enrolled >10,000 participants using standardized methodology and photographic grading for AMD and risk factor assessment^28^. In this report, we performed case-control comparison of risk factors between 456 patients with nAMD and 1,824 controls free of AMD who were matched for age, gender and ethnicity.

## Methods

### Study Population

We performed a case-control study to determine risk factors of nAMD using data from 456 cases with nAMD enrolled in the Asian AMD Phenotyping Study and 1,824 age and gender-matched controls free of AMD enrolled in the Singapore Epidemiology of Eye Diseases program. These studies received approval from the Singhealth Institutional Review Board or the National Healthcare Group Domain Specific Review Board (Domain A) and were conducted in accordance with the Declaration of Helsinki (protocol number R697/47/2009, R341/34/2003, R498/47/2006 and DSRB2011/00319). All participants provided written informed consent.

#### nAMD Cases

The Asian AMD Phenotyping Study is a prospectively planned, cohort study to investigate prospectively a consecutive series of treatment-naıve Asian patients with exudative maculopathy secondary to neovascular AMD. Detailed study protocol has been published previously^1^,^16^,^28^,^39^. All patients provided written informed consent to participate in this research. Consecutive patients were recruited from the retinal clinics of the Singapore National Eye Centre and the Tan Tock Seng Hospital. Recruitment started on March 01 2010 and is still ongoing.

#### Population Controls

We selected age-(within 5 years), gender- and ethnicity-matched controls from the Singapore Epidemiology of Eye Disease Study program which comprises 3 population-based studies, with the baseline examinations conducted from 2004 through 2011: the Singapore Malay Eye Study (2004–2006)^9^,^40^, the Singapore Indian Eye Study (2007–2009)^41^, and the Singapore Chinese Eye Study (2009–2011)^41^. The detailed methodology has been described elsewhere. For each case we selected 4 controls matched for age, gender and ethnicity. All three population studies were conducted at the Singapore Eye Research Institute.

### Clinical Evaluation and AMD grading

#### nAMD cases

Each patient had an interview, systemic examination, and laboratory investigations to determine socioeconomic, ocular, and systemic risk factors following the same protocol as the Singapore Epidemiology of Eye Disease Study program. The examination procedures were performed at baseline visit and include measurements of height, weight, blood pressure and pulse rate, followed by a comprehensive ocular examination. This included best-corrected visual acuity (BCVA, measured in Snellen and converted to LogMAR for analysis), dilated fundus examination, fluorescein and ICGA, fundus autofluorescence (FAF) imaging, color fundus photography and optical coherence tomography (OCT) according to a standardized protocol at baseline. Fundus photography was performed using a digital mydriatic retinal camera (TRC-50X/IMAGEnet 2000, Topcon, Tokyo, Japan). Auto refraction is performed using an auto-refractor (Canon RK-5 Auto Ref-Keratometer, Canon Inc. Ltd, Tokyo, Japan). Ocular biometry is measured using the IOL Master (Carl Zeiss; Meditec AG, Jena, Germany). nAMD was diagnosed clinically and confirmed by ocular imaging results which were graded by retinal specialists. Subtypes of typical AMD and polypoidal choroidal vasculopathy (PCV) were diagnosed according to ICGA features. The diagnosis of definitive PCV lesions was based on the Japanese Study Group guidelines^42^. Systolic and diastolic blood pressure were measured in a standard manner after 5 minutes of rest with a digital automatic blood pressure monitor.

#### Population controls

All participants had an interview, systemic examination, and laboratory investigations to determine socioeconomic, ocular, and systemic risk factors. We used a digital fundus camera (Canon CR-DFi with 10D SLR digital camera back; Canon, Tokyo, Japan) to capture color photographs of each eye after pupil dilation. The Centre for Vision Research, University of Sydney, performed AMD grading using a modification of the Wisconsin Age-Related Maculopathy Grading System^43^ which defines early AMD as either soft indistinct or reticular drusen or both soft, distinct drusen plus retinal pigment epithelium abnormalities; late AMD is defined as having either nAMD or geographic atrophy. Subjects free of any stage of AMD were selected as controls for this study. We obtained AMD grading from right eyes, unless the parameter studied was ungradable, in which case we obtained grading from the left eye. From the 9,799 participants for whom AMD was gradable from fundus photographs, early AMD was present in 588 and late AMD in 63 participants. Controls for the current study were selected from the remaining participants who had no AMD.

### Systemic and Ocular Risk Factors and Definitions

A detailed interviewer-administered questionnaire is used to collect information about medical history (including hypertension, diabetes, angina, myocardial infarction and stroke), cigarette smoking (defined as current, past and never). The questionnaire is administered in English or translated into Chinese (Mandarin), Tamil or Malay and back-translated into English. We defined risk factors using similar definitions for the purpose of this analysis. We defined age as the age at examination. We categorized cigarette smoking into current smokers, former smokers or nonsmokers. We defined hypertension as a recorded systolic blood pressure ≥140 mmHg or diastolic BP ≥ 90 mmHg or self-reported physician-diagnosed hypertension. We defined diabetes mellitus as random glucose of 11.1 mmol/L or more, a self-reported physician diagnosis of diabetes, or use of glucose-lowering medication. We collected a 4.0 ml non-fasting sample of venous blood from each participant to determine the levels of lipid, glucose, glycated hemoglobin (HbA1c) and creatinine.

### Genotype

We extracted DNA from venous blood sample from all participants. Due to funding restrictions, only a subgroup (150 cases, 1,267 controls, all Chinese patients) had been genotyped so far, using Illumina Human OmniExpress or Human Hap610-Quad Beadchip. For the current analysis, we included 2 major AMD associated SNPs, rs800292 from *CFH* and rs10490924 from *ARMS2*. In view of our recent discovery of Asian specific polymorphism in CETP rs2303790 associated with AMD, we also included SNPS in *CETP* (rs3764261 and rs2303790 D442G) in our analysis^33^,^34^,^35^,^36^,^37^.

### Statistical Analysis

We performed all statistical analyses using Stata 11.1 (College Station, Texas 77845 USA). We used means and standard deviations to describe characteristics of the study population. We analyzed the relationship between AMD and risk factors using logistic regression models to estimate the odds ratios (ORs) with 95% confidence intervals (CIs). We adjusted for age and gender in the first model. Additionally, for the multiple-adjusted analysis, we included risk factors with P < 0.3 in age- and gender-adjusted model and also well-established risk factors (i.e. smoking, diabetes and hypertension status).

## Results

### nAMD vs Controls

In total, 456 cases with nAMD (215 typical AMD and 241 PCV) and 1824 controls were included. The characteristics of participants and risk factors have been summarized in Table 1. In the nAMD group, the mean age was 69.4 ± 9.36 years, and the majority was male (62.5%) and Chinese (93.2%). The control group was closely matched, with mean age of 64.4 ± 8.53 years, 62.7% were male and 93.2% were Chinese.

In the univariate analysis (Table 1), patients with nAMD were older despite age-matching (69.4 vs 64.4 years, p < 0.001), had significantly higher body mass index (BMI) (24.4 vs 23.9, p = 0.012), lower total cholesterol (5.09 vs 5.37 mmol/L, p < 0.001), lower LDL-cholesterol (3.09 vs 3.22 mmol/L, p = 0.048) and higher HDL-cholesterol (1.38 vs 1.27 mmol/L, p < 0.001), shorter axial length (23.53 vs 23.94mm, p < 0.001) and were more likely to be smokers (41.9% vs 33.6%, p = 0.003) and pseudophakic (28.3 vs 14.2%, p < 0.001) and have a history of cardiovascular disease (13.6% vs 10.1%, p = 0.039). After adjusting for age and gender, factors that remained significantly associated with nAMD were older age, higher BMI, current smoking, higher HDL-cholesterol and pseudophakia. (Table 2) Hypertension total cholesterol and axial length were inversely associated with nAMD. In the multiple-adjusted model, increasing age, male gender, higher BMI, higher HDL-cholesterol, shorter axial length and pseudophakia remained significant risk factors for nAMD. There was a trend towards an association with smoking but the association was not significant after adjusting for other risk factors.

Patients with nAMD were further divided into those with typical AMD and PCV subtypes according to angiographic features. Risk factors for each subtype were further compared with control group. (Table 2) Risk factors common for both typical AMD and PCV included increasing age, higher BMI and higher HDL-cholesterol. Shorter axial length was associated with increased risk of PCV and showed a trend towards similar association with typical AMD. In addition, pseudophakia was associated with typical AMD but not PCV. Female gender and hypertension were inversely associated with typical AMD but not PCV.

### Influence of genetic factors

In the subgroup of patients with genotype information (64 typical AMD, 86 PCV, 1,267 controls), both *CFH* rs800292 (OR 0.55, p = 0.009) and *ARMS2* rs10490924 (OR 2.70, p < 0.001) were significantly associated with nAMD, as were increasing age and past smoking. (Table 3) When patients were further divided into typical AMD and PCV subgroups, *ARMS2* remained significantly associated with both subtypes (OR 3.57, p = 0.001 for typical AMD; 2.54, p < 0.001 for PCV), whereas *CFH* rs800292 A allele also showed a marginal association with each subgroup. (typical AMD OR 0.49, p = 0.085; PCV OR 0.62, p = 0.062)

### Typical AMD vs PCV Subtypes

Patients with PCV were significantly younger (68.3 vs 70.7 years, p = 0.008) and had better presenting vision (0.76 vs 1.00, p < 0.001). (Table 4) However, after adjusting for age and gender, none of the risk factors were significantly different between the two subtypes.

## Discussion

The current understanding of risk factors for nAMD in Asians is limited. Previous reports have been somewhat inconsistent, and most studies conducted in population-based settings were limited by few late or nAMD cases. The Beijing Eye Study and the Shihpai Eye study both failed to identify any systemic risk factors for late AMD^7^,^8^. In the Singapore Epidemiology of Eye Disease program which enrolled 10,033 persons in Singapore, only 63 individuals had late AMD. Risk factors for late AMD included older age, smoking >5 packs per week and presence of chronic kidney disease^14^. In the current study, we have the unique opportunity to compare risk factors of nAMD using a large prospectively recruited clinical cohort of patients and age-, gender- and ethnicity-matched controls from a population study. Both clinical cases and population controls were examined using a standardized protocol and questionnaire.

Similar to previous studies in Asians, we report a significant male predominance (62.5%) in patients with nAMD. In terms of ethnicity, there appears to be over-representation of Chinese (93.2%) compared to that expected in the Singapore general population in which 76.2% are Chinese^44^. Systemic risk factors for nAMD in this Asian population included older age, higher BMI and higher HDL-cholesterol. Previous studies which evaluated the association between AMD and HDL have been somewhat inconsistent, with raised HDL cholesterol levels reported to be protective in some studies while others reported increased AMD risk^18^,^38^,^45^. The association between diabetes mellitus and AMD has been inconsistent. Our results did not demonstrate any associations between nAMD and diabetes mellitus or hypertension. Most recently, Hahn *et al*. used Medicare claims database reported a hazard ratio of more than two fold greater for wet AMD in individuals with proliferative diabetic retinopathy than in non-diabetic controls, while individuals who had diabetes without retinopathy had no increased incidence of AMD^46^. In the current study, however, we did not grade the stage of diabetic retinopathy. We note a high proportion of controls with hypertension (70.3%) and diabetes (39.6%), which may reflect the common use of hypertensive medication, in particular, in this population with mean age of 64 years. The high prevalence of these conditions in the control group may have limited the ability to detect differences between cases and controls. Smoking was associated with increased risk of nAMD in the age and gender adjusted model. However, after adjusting for multiple risk factors, the association did not reach statistical significance, mainly due to wide confidence intervals. However the odds ratios remained >1.0, which suggests the lack of association demonstrated is likely due to limitation in sample size. Finally, the association between increased BMI and AMD is mild (OR 1.08) despite statistical significance.

Ocular risk factors associated with nAMD included previous cataract surgery and shorter axial length. We and others have previously reported hyperopia as a risk factor for any AMD or early AMD in population studies^8^,^26^,^27^,^47^. In this study, we found further support of this association by demonstrating that shorter axial length was associated with increased risk of nAMD. The association between cataract surgery and AMD has been inconsistently described. Increased risk for late AMD has been associated with cataract surgery in several large population studies^48^,^49^,^50^,^51^. Proposed mechanisms include exposure to blue light and increased inflammation^52^. However, other studies have failed to detect an association between cataract surgery and AMD progression, most notably in the large Age-related Eye diseases Study (AREDS)^53^ and also recently in the Korean National Health and Nutrition Examination Survey (KNHANES)^54^,^55^. A recent systematic review also concluded cataract surgery did not increase the risk of progression to exudative AMD^56^. Interesting, we only demonstrated significant association between cataract surgery with typical AMD but not with PCV. The relationship between cataract surgery and AMD this requires further evaluation with longer follow-up.

We compared risk factors between typical AMD and PCV subtypes, and report that patients with PCV were significantly younger and presented with better vision^1^,^29^,^57^. These findings are in keeping with previous reports. However, after adjusting for age and gender, none of the risk factors studied were significantly different between the two groups. There are few studies to compare with. Sakurada *et al*. compared systemic risk factors between typical AMD and PCV patients and reported diabetes mellitus and end-stage renal disease were more prevalent in patients with typical AMD than those with PCV^30^. Increased serum vascular endothelial growth factor and complement activation have been proposed as possible common mechanisms between these systemic disorders and AMD. A similar study by Ueta *et al*. reported similar background factors in patients with typical AMD and PCV, apart from higher prevalence of diabetes mellitus and history of central serous chorioretinopathy^31^. We note that a significantly higher proportion of subjects had diabetes in the current study (39.6% in controls, 40.7% in typical AMD and 37.0% in PCV) compared to that reported in Sakurada’s series (22.6% in typical AMD and 12.2% in PCV) and Ueta’s series (24.7% typical AMD, 13.0% PCV). Although the PCV group had lower creatinine than the typical AMD group, the difference was no longer significant after adjusting for age, gender and ethnicity.

We included analysis of a subgroup of participants in whom genetic data was available. However, since genetic testing was not performed in all participants due to funding constraints, the strength of this sub-analysis may be limited. Nevertheless, we demonstrated an association between *CFH* and *ARMS2* SNPs tested with nAMD and AMtypical AMD subgroup. There was also a marginal association with PCV subgroup, which was likely reflection of limited sample size. We also noted that genetic factors appear to have larger effects as compared to other non-genetic factors, as demonstrated by higher odds ratios. There have been increasing interests in the potential role of HDL metabolism in AMD pathogenesis after several genes involved in HDL metabolism have been associated with AMD. We recently identified a novel missense mutation in the *CETP* gene. The mutant G allele is associated with elevated HDL cholesterol and increased risk of AMD^36^. Results from the current study which showed that higher HDL is associated with nAMD is somewhat consistent with the previous report, although the association was independent of the two CETP SNPs studied. The association between HDL and PCV compared to typical AMD is less clear. Zhang *et al*. reported that serum HDL cholesterol levels were significantly lower in patients with PCV (1.36 ± 0.27 mmol/L) and typcial AMD (1.32 ± 0.33 mmol/L) compared to controls (1.46 ± 0.37 mmol/L). Results from the current study showed contradictory results, with higher HDL in typical AMD (1.42 ± 0.48 mmol/L), followed by PCV (1.34 ± 0.34 mmol/L) and lowest in controls (1.27 ± 0.39 mmol/L) These results further support the need to study the role of HDL metabolism perturbation in AMD, including PCV.

The strengths of the current study include the large sample size of prospectively recruited patients and controls examined using a common protocol and questionnaire to collect demographic data specifically designed for risk factor analysis. Another strength is the inclusion of patients with typical AMD and PCV subtypes who were all categorized according to fluorescein and indocyanine green angiography findings using a standardized protocol for image acquisition and grading. There are weaknesses to mention. Due to the predominance of patients of Chinese ethnicity, our results are largely driven by this ethnic group. Despite our attempt to match for age, gender and ethnicity, the control group was still younger than the typical AMD group, due to limited number of older subjects in the population study. The nAMD patients were recruited from clinic and therefore did not receive subjective refraction at presentation. Therefore we used axial length as a surrogate for assessment of refractive status. Lack of fasting may have affected results of lipid parameters. Another limitation is that only a subgroup of subjects had genotype data available due to funding restrictions. Finally due to limited number of patients with advanced disease in population studies and the lack of systematic risk factor data collection from clinic-based cohorts, our unique study design compares subjects from hospital based cohort with those from a population based study. Thus despite the carefully designed and standardized protocol, we acknowledge there remains inherent differences in these cohorts and the possibility of selection bias. Similarly, despite recruiting the patients from the two largest public hospitals, there may still be selection bias such as differences in catchment areas.

In summary, we conducted a case-control study of predominantly Chinese patients with nAMD and controls in Singapore and report that older age, male gender, body mass index, HDL-cholesterol, shorter axial length and previous cataract surgery were significant risk factors for nAMD. Patients with PCV were younger than those with typical AMD, and presented with better vision. However, other demographic and genetic factors were similar between these two subtypes. Our data add to the concept that while there are marked differences in clinical presentation between typical AMD and PCV, underlying risk factors appears to be similar.

## Additional Information

**How to cite this article**: Cheung, C. M. G. *et al*. Systemic, Ocular and Genetic Risk Factors for Age-related Macular Degeneration and Polypoidal Choroidal Vasculopathy in Singaporeans. *Sci. Rep.* **7**, 41386; doi: 10.1038/srep41386 (2017).

**Publisher's note:** Springer Nature remains neutral with regard to jurisdictional claims in published maps and institutional affiliations.

## Figures and Tables

**Table 1:** Comparison of baseline demographics between patients with neovascular Age-related Macular Degeneration (nAMD) and age and gender matched controls.

	Controls (N = 1824)	nAMD (N = 456)	P value
Age (years), Mean (SD)	64.4 (8.53)	69.4 (9.36)	<0.001
Gender, male (%)	62.7	62.5	0.931
Race			0.936
Chinese (%)	93.2	93.2	
Malay (%)	5.0	4.82	
Indian (%)	1.8	1.97	
Presenting Vision ± SD (LogMAR)	0.33 (0.33)	0.87 (0.63)	<0.001
BMI ± SD (kg/m^2^), Mean (SD)	23.9 (3.67)	24.4 (4.04)	0.012
Hypertension (%)	70.3	71.9	0.496
Creatinine ± SD (mmol/L), Mean (SD)	82.63 (45.6)	83.25 (32.9)	0.799
Smoking status			0.003
Nonsmoker (%)	66.4	58.1	
Past smoker (%)	17.9	24.3	
Current smoker (%)	15.7	17.6	
Cardiovascular Disease (%)	10.1%	13.6%	0.039
Stroke (%)	3.18%	5.04	0.069
Diabetes (%)	39.6	38.7	0.730
Glucose ± SD (mmol/L)	6.63 (3.03)	6.57 (2.29)	0.836
Hba1c ± SD (%)	6.14 (0.98)	6.09 (0.86)	0.306
Total Cholesterol ± SD (mmol/L)	5.37 (1.10)	5.09 (1.11)	<0.001
LDL-cholesterol ± SD (mmol/L)	3.22 (0.92)	3.09 (0.91)	0.048
HDL-cholesterol ± SD (mmol/L)	1.27 (0.39)	1.38 (0.41)	<0.001
Axial Length ± SD (mm)	23.94 (1.41)	23.53 (1.74)	<0.001
Pseudophakia (%)	14.2	28.3	<0.001

SD standard deviation; BMI body mass index; LDL-cholesterol Low density Lipoprotein- cholesterol; HDL-cholesterol High density Lipoprotein cholesterol.

**Table 2:** Risk Factors for neovascular Age-related macular degeneration.

	Neovascular AMD (n = 456) vs Controls (n = 1,824)				Typical AMD (n = 215) vs Controls (n = 1,824)				PCV (n = 241) vs Controls (n = 1,824)			
	Age and gender adjusted OR (95% CI)	p	Multiple Adjusted# OR (95% CI)	p	Age and gender adjusted OR (95%)	p	Multiple Adjusted* OR (95%)	p	Age and gender adjusted OR (95%)	p	Multiple Adjusted# OR (95%)	p
Age, years	1.07 (1.06, 1.08)	<0.001	1.07 (1.05, 1.10)	<0.001	1.09 (1.07, 1.11)	<0.001	1.10 (1.06, 1.13)	<0.001	1.06 (1.04, 1.07)	<0.001	1.05 (1.02, 1.08)	0.001
Gender, female	0.87 (0.70, 1.08)	0.199	0.55 (0.34, 0.86)	0.010	0.76 (0.56, 1.03)	0.079	0.48 (0.28, 0.83)	0.009	0.92 (0.69, 1.22)	0.552	0.56 (0.30, 1.03)	0.064
Race												
Chinese	Reference	—	—	—	Reference		—	—	Reference		—	—
Malay	1.02 (0.62, 1.66)	0.944	—	—	1.03 (0.53, 1.99)	0.940	—	—	0.94 (0.49, 1.80)	0.853	—	—
Indian	1.33 (0.61, 2.89)	0.477	—	—	1.30 (0.44, 3.87)	0.3.6	—	—	1.32 (0.50, 3.48)	0.579	—	—
Body Mass Index (BMI)	1.06 (1.02, 1.09)	<0.001	1.08 (1.03, 1.13)	0.002	1.06 (1.02, 1.11)	0.004	1.09 (1.02, 1.16)	0.014	1.05 (1.01, 1.09)	0.013	1.07 (1.01, 1.14)	0.027
Hypertension	0.72 (0.57, 0.93)	0.011	0.73 (0.49, 1.10)	0.131	0.71 (0.50, 1.01)	0.054	0.49 (0.28, 0.84)	0.010	0.73 (0.53, 1.00)	0.047	0.91 (0.54, 1.53)	0.728
Creatinine	1.00 (0.99, 1.00)	0.203	1.00 (0.99, 1.00)	0.235	1.00 (1.00, 1.00)	0.686	—	—	1.00 (0.99, 1.00)	0.118	0.99 (0.99, 1.00)	0.200
Smoking status												
Nonsmoker	Reference		Reference		Reference		—	—	Reference		Reference	
Current smoker	1.51 (1.08, 2.11)	0.015	1.37 (0.83, 2.28)	0.222	1.25 (0.78, 2.01)	0.349			1.77 (1.17, 2.70)	0.007	1.33 (0.69, 2.55)	0.392
Past smoker	1.35 (0.99, 1.83)	0.056	1.28 (0.82, 2.00)	0.274	1.14 (0.75, 1.73)	0.544	—	—	1.53 (1.03, 2.28)	0.036	1.47 (0.84, 2.56)	0.180
Current and past smoker												
No	Reference		Reference		Reference		—	—	Reference		Reference	
Yes	1.35 (0.99, 1.83)	0.058	1.24 (0.77, 2.00)	0.366	1.19 (0.77, 1.86)	0.435			1.50 (1.02, 2.21)	0.038	1.15 (0.62, 2.10)	0.662
Cardiovascular disease	1.06 (0.75, 1.49)	0.734	—	—	1.08 (0.69, 1.70)	0.732	—	—	1.00 (0.64, 1.57)	0.997	—	—
Stroke	1.13 (0.66, 1.93)	0.665	—	—	0.85 (0.39, 1.86)	0.692	—	—	1.38 (0.72, 2.66)	0.331	—	—
Diabetes	0.87 (0.69, 1.09)	0.216	1.17 (0.76, 1.82)	0.476	0.94 (0.69, 1.27)	0.680	—	—	0.81 (0.61, 1.08)	0.156	1.20 (0.67, 2.13)	0.542
Glucose	0.99 (0.92, 1.06)	0.765	—	—	1.03 (0.94, 1.12)	0.536	—	—	0.96 (0.86, 1.06)	0.397	—	—
HbA1c	0.92 (0.80, 1.05)	0.203	0.86 (0.66, 1.13)	0.277	0.97 (0.81, 1.16)	0.734	—	—	0.87 (0.73, 1.05)	0.154	0.71 (0.47, 1.07)	0.102
Total Cholesterol	0.87 (0.78, 0.97)	0.012	0.87 (0.73, 1.03)	0.108	0.89 (0.76, 1.03)	0.126	0.88 (0.68, 1.13)	0.322	0.85 (0.73, 0.98)	0.026	0.85 (0.68, 1.05)	0.137
LDL-cholesterol	0.99 (0.83, 1.16)	0.874	—	—	1.08 (0.86, 1.35)	0.513	—	—	0.92 (0.74, 1.15)	0.461	—	—
HDL-cholesterol	1.95 (1.36, 2.77)	<0.001	2.51 (1.63, 3.86)	<0.001	2.30 (1.44, 3.69)	0.001	3.36 (1.91, 5.91)	<0.001	1.59 (0.98, 2.57)	0.062	1.84 (1.02, 3.35)	0.045
Axial Length	0.84 (0.77, 0.92)	<0.001	0.84 (0.74, 0.97)	0.015	0.88 (0.80, 0.98)	0.018	0.84 (0.70, 1.01)	0.064	0.78 (0.69, 0.89)	<0.001	0.83 (0.71, 0.99)	0.034
Pseudophakia	1.43 (1.09, 1.87)	0.010	1.69 (1.13, 2.55)	0.011	1.56 (1.09, 2.21)	0.014	2.20 (1.28, 3.80)	0.005	1.30 (0.91, 1.84)	0.151	1.53 (0.89, 2.63)	0.128

^*^Adjusted for age, gender, BMI, hypertension, total cholesterol, HDL, axial length and cataract surgery.

^#^Adjusted for age, gender, BMI, hypertension, creatinine, smoking status, diabetes, HbA1c, total cholesterol, HDL, axial length and cataract surgery.

Lipoprotein- cholesterol; HDL-cholesterol High density Lipoprotein cholesterol; OR SD standard deviation; BMI body mass index; LDL-cholesterol Low density; OR Odds Ratio; CI Confidence intervals.

**Table 3:** Genetics and demographic risk factors for neovascular Age-related macular degeneration.

	Neovascular AMD (n = 150) vs Controls (n = 1,267)				Typical AMD (n = 64) vs Controls (n = 1,267)				PCV (n = 86) vs Controls (n = 1,267)			
	Age and gender adjusted OR (95% CI)	p	Multiple Adjusted^#^ OR (95% CI)	p	Age and gender adjusted OR (95%)	p	Multiple Adjusted* OR (95%)	p	Age and gender adjusted OR (95%)	p	Multiple Adjusted# OR (95%)	p
Age, years	1.07 (1.05, 1.09)	<0.001	1.07 (1.03, 1.11)	<0.001	1.09 (1.06, 1.13)	<0.001	1.09 (1.01, 1.17)	0.021	1.05 (1.03, 1.08)	<0.001	1.07 (1.03, 1.12)	0.001
Gender, female	0.89 (0.62, 1.27)	0.517	0.80 (0.39, 1.66)	0.552	0.68 (0.39, 1.18)	0.170	0.41 (0.12, 1.40)	0.154	1.05 (0.67, 1.66)	0.827	0.92 (0.39, 2.18)	0.851
Body Mass Index (BMI)	1.06 (1.01, 1.12)	0.014	1.04 (0.96, 1.12)	0.364	1.11 (1.03, 1.19)	0.006	1.13 (0.99, 1.30)	0.081	1.04 (0.97, 1.10)	0.279	1.00 (0.90, 1.10)	0.939
Hypertension	0.92 (0.61, 1.38)	0.675	—		0.72 (0.40, 1.30)	0.272	0.36 (0.12, 1.08)	0.068	1.11 (0.66, 1.89)	0.691	—	—
Creatinine	1.00 (1.00, 1.01)	0.554	—		1.00 (1.00, 1.01)	0.150	1.01 (0.99, 1.02)	0.527	1.00 (0.99, 1.01)	0.576	—	—
Smoking status												
Nonsmoker	Reference		Reference		Reference				Reference		Reference	
Current smoker	1.64 (0.94, 2.86)	0.084	1.73 (0.75, 4.00)	0.199	1.17 (0.48, 2.85)	0.734	—	—	2.06 (1.03, 4.10)	0.040	2.12 (0.82, 5.47)	0.120
Past smoker	1.63 (1.00, 2.65)	0.052	2.22 (1.05, 4.68)	0.037	1.42 (0.71, 2.85)	0.327	—	—	1.77 (0.93, 3.37)	0.081	2.89 (1.23, 6.79)	0.015
Current and past smoker							—	—				
No	Reference		Reference		Reference				Reference		Reference	
Yes	1.37 (0.81, 2.30)	0.242	1.30 (0.60, 2.81)	0.510	1.02 (0.44, 2.38)	0.962			1.67 (0.88, 3.16)	0.114	1.44 (0.61, 3.41)	0.410
Cardiovascular disease	0.81 (0.42, 1.55)	0.523	—		1.23 (0.54, 2.76)	0.623	—	—	0.48 (0.17, 1.37)	0.170	0.16 (0.02, 1.31)	0.088
Stroke	0.57 (0.17, 1.92)	0.362	—		0.80 (0.18, 3.55)	0.772	—	—	0.35 (0.05, 2.64)	0.309	—	
Diabetes	0.82 (0.57, 1.17)	0.277	1.00 (0.46, 2.16)	1.000	1.08 (0.64, 1.83)	0.762	—	—	0.66 (0.41, 1.06)	0.088	0.64 (0.25, 1.61)	0.344
Glucose	0.92 (0.83, 1.03)	0.159	0.96 (0.81, 1.13)	0.589	0.95 (0.80, 1.13)	0.544	—	—	0.91 (0.79, 1.05)	0.195	0.96 (0.79, 1.16)	0.665
HbA1c	0.84 (0.67, 1.06)	0.147	0.82 (0.45, 1.49)	0.520	0.89 (0.65, 1.23)	0.490	—	—	0.81 (0.60, 1.11)	0.187	1.00 (0.51, 1.95)	1.000
Total Cholesterol	1.00 (0.85, 1.18)	0.963	—		1.00 (0.79, 1.29)	0.956	—	—	1.01 (0.82, 1.25)	0.916	—	—
LDL-cholesterol	1.08 (0.84, 1.39)	0.565	—		1.26 (0.85, 1.86)	0.246	1.66 (1.01, 2.73)	0.046	1.00 (0.73, 1.37)	0.987	—	—
HDL-cholesterol	1.89 (1.11, 3.24)	0.020	1.79 (0.97, 3.31)	0.062	2.43 (1.06, 5.57)	0.035	3.61 (1.46, 8.94)	0.006	1.63 (0.81, 3.27)	0.170	1.09 (0.46, 2.59)	0.839
Axial Length	0.85 (0.76, 0.96)	0.009	0.80 (0.65, 0.99)	0.039	0.85 (0.75, 0.98)	0.019	0.76 (0.53, 1.09)	0.135	0.82 (0.68, 0.99)	0.039	0.81 (0.64, 1.04)	0.101
Pseudophakia	1.37 (0.88, 2.12)	0.165	1.16 (0.55, 2.43)	0.694	1.51 (0.81, 2.82)	0.193	2.68 (0.75, 9.54)	0.127	1.24 (0.70, 2.21)	0.456	—	
CFH (rs800292_A)	0.63 (0.48, 0.82)	0.001	0.55 (0.36, 0.86)	0.009	0.69 (0.46, 1.01)	0.059	0.49 (0.21, 1.10)	0.085	0.59 (0.42, 0.83)	0.003	0.62 (0.37, 1.02)	0.062
ARMS2 (rs10490924_T)	2.20 (1.69, 2.85)	<0.001	2.70 (1.77, 4.11)	<0.001	2.64 (1.77, 3.94)	<0.001	3.57 (1.63, 7.83)	0.001	1.99 (1.44, 2.76)	<0.001	2.53 (1.56, 4.11)	<0.001
CETP (rs3764261_A)	1.04 (0.75, 1.45)	0.793	—	—	1.00 (0.61, 1.64)	0.994	—	—	1.07 (0.71, 1.62)	0.741	—	—
D442G (rs2303790_G)	1.23 (0.61, 2.49)	0.559	—	—	1.39 (0.53, 3.65)	0.503	—	—	1.12 (0.44, 2.89)	0.807	—	—

^#^Adjusted for age, gender, BMI, diabetes, smoking, glucose, Hba1c, HDL cholesterol, axial length, cataract surgery, CFH and ARMS2.

^*^Adjusted for age, gender, BMI, hypertension, creatinine, LDL cholesterol, HDL cholesterol, axial length, cataract surgery, CFH and ARMS2.

SD standard deviation; BMI body mass index; LDL-cholesterol Low density Lipoprotein- cholesterol; HDL-cholesterol High density Lipoprotein cholesterol. **Adjusted for age, gender, BMI, diabetes, cardiovascular disease, smoking, glucose, HbA1c, HDL cholesterol, axial length, CFH and ARMS2.

**Table 4:** Comparison of baseline demographics between patients with typcial Age-related Macular Degeneration (typical AMD) and polypoidal choroidal vasculopathy (PCV).

	Baseline Characteristics			Demographic risk factor analysis PCV (n = 241) vs typical AMD (n = 215)				Genetic and demographic risk factor analysis PCV (n = 86) vs typical AMD (n = 64)			
	AMD (N = 215)	PCV (N = 241)	P	Age and gender adjusted OR (95% CI)	p	Multiple Adjusted* OR (95% CI)	p	Age and gender adjusted OR (95% CI)	p	Multiple Adjusted# OR (95% CI)	p
Age (years)	70.7 (10.15)	68.3 (8.47)	0.008	0.97 (0.95, 0.99)	0.009	0.96 (0.91, 1.00)	0.064	0.97 (0.94, 1.01)	0.097	0.97 (0.93, 1.01)	0.104
Gender, female (%)	36.3	38.6	0.611	1.06 (0.72, 1.56)	0.762	0.69 (0.22, 2.18)	0.528	1.38 (0.69, 2.72)	0.361	1.09 (0.40, 2.99)	0.867
Race			0.956								
Chinese (%)	93.0	93.4		Reference		—		—	—	—	—
Malay (%)	5.12	4.56		0.78 (0.32, 1.85)	0.557	—		—	—	—	—
Indian (%)	1.86	2.07		1.00 (0.26, 3.84)	0.999	—		—	—	—	—
Presenting Vision ± SD (LogMAR)	1.00 (0.64)	0.76 (0.60)	<0.001	NA		NA		—	—	—	—
BMI ± SD (kg/m^2^)	24.4 (4.32)	24.4 (3.79)	0.973	0.99 (0.94, 1.04)	0.668	—		0.93 (0.85, 1.02)	0.131	0.94 (0.85, 1.04)	0.229
Hypertension (%)	73.4	70.6	0.506	1.00 (0.65, 2.04)	0.985	—		1.62 (0.73, 3.58)	0.232	2.03 (0.77, 5.34)	0.153
Creatinine ± SD (mmol/L)	86.7 (39.6)	80.0 (24.7)	0.044	0.99 (0.99, 1.00)	0.134	0.99 (0.98, 1.00)	0.316	0.99 (0.98, 1.00)	0.063	0.99 (0.98, 1.00)	0.167
Smoking status			0.508								
Nonsmoker (%)	60.3	56.1		Reference		Reference		Reference		Reference	
Past smoker (%)	24.3	24.3		1.30 (0.75, 2.26)	0.350	0.86 (0.29, 2.53)	0.788	1.16 (0.46, 2.92)	0.755	1.19 (0.42, 3.36)	0.745
Current smoker (%)	15.3	19.6		1.49 (0.81, 2.75)	0.197	1.33 (0.35, 5.03)	0.675	1.93 (0.63, 5.89)	0.246	1.25 (0.36, 4.27)	0.726
Current and past smoker			0.260								
No	84.7	80.4		Reference		Reference		Reference		Reference	
Yes	15.3	19.6		1.32 (0.76, 2.30)	0.323	1.43 (0.42, 4.86)	0.571	1.81 (0.65, 5.06)	0.260	1.15 (0.37, 3.53)	0.812
Cardiovascular disease (%)	15.1	12.3	0.416	0.82 (0.45, 1.49)	0.517	—		0.37 (0.10, 1.37)	0.136	0.41 (0.10, 1.68)	0.216
Stroke (%)	4.35	5.63	0.559	1.52 (0.59, 3.87)	0.385	—		0.43 (0.04, 5.08)	0.506	—	—
Diabetes (%)	40.7	37.0	0.434	0.87 (0.59, 1.29)	0.488	—		0.63 (0.32, 1.26)	0.194	0.62 (0.27, 1.39)	0.244
Glucose ± SD (mmol/L)	6.91 (2.50)	6.36 (2.14)	0.209	0.91 (0.77, 1.07)	0.265	0.92 (0.76, 1.12)	0.416	0.97 (0.77, 1.23)	0.829	—	—
Hba1c ± SD (%)	6.13 (0.92)	6.06 (0.79)	0.455	0.92 (0.73, 1.17)	0.506	—		0.95 (0.62, 1.46)	0.823	—	—
Total Cholesterol ± SD (mmol/L)	5.07 (1.15)	5.11 (1.07)	0.722	0.95 (0.79, 1.16)	0.628	—		1.04 (0.77, 1.42)	0.784	—	—
LDL-cholesterol ± SD (mmol/L)	3.11 (0.98)	3.07 (0.84)	0.746	0.88 (0.64, 1.19)	0.401	—		0.80 (0.44, 1.46)	0.467	—	—
HDL-cholesterol ± SD (mmol/L)	1.42 (0.48)	1.34 (0.34)	0.125	0.54 (0.26, 1.12)	0.100	0.79 (0.30, 2.05)	0.630	0.58 (0.20, 1.67)	0.315	—	—
Axial Length ± SD (mm)	23.6 (2.31)	23.5 (1.07)	0.816	0.97 (0.85, 1.10)	0.639	—		1.04 (0.90, 1.20)	0.622	—	—
Pseudophakia (%)	32.6	24.5	0.056	0.81 (0.52, 1.27)	0.358	—		0.77 (0.35, 1.71)	0.524	—	—
CFH (rs800292_A)	—	—	—	—		—		0.84 (0.49, 1.45)	0.541	—	—
ARMS2 (rs10490924_T)	—	—	—	—		—		0.80 (0.50, 1.28)	0.359	—	—
CETP (rs3764261_A)	—	—	—	—		—		1.08 (0.59, 1.99)	0.799	—	—
D442G (rs2303790_G)	—	—	—	—		—		0.80 (0.22, 2.96)	0.740	—	—

^*^Adjusted for age, gender, smoking, creatinine, glucose and HDL cholesterol.

^#^Adjusted for age, gender, BMI, hypertension, diabetes, smoking, creatinine and cardiovascular disease.

Genotype analysis was available in a subgroup of patients. SD standard deviation; BMI body mass index; LDL-cholesterol Low density Lipoprotein- cholesterol; HDL-cholesterol High density Lipoprotein cholesterol; OR Odds ratio, CI confidence intervals.
